# Antitumor Effect of ^131^I-Labeled Anti-VEGFR2 Targeted Mesoporous Silica Nanoparticles in Anaplastic Thyroid Cancer

**DOI:** 10.1186/s11671-019-2924-z

**Published:** 2019-03-14

**Authors:** Ruiguo Zhang, Yueqian Zhang, Jian Tan, Hanjie Wang, Guizhi Zhang, Ning Li, Zhaowei Meng, Fuhai Zhang, Jin Chang, Renfei Wang

**Affiliations:** 1 Department of Nuclear Medicine, Tianjin Medical University General Hospital, No. 154 Anshan Road, Heping District, Tianjin, 300052 China; 2 School of Life Sciences, Tianjin University, Tianjin Engineering Center of Micro-Nano Biomaterials and Detection-Treatment Technology, Tianjin, 300072 China

**Keywords:** Anaplastic thyroid cancer, ATC, Vascular endothelial growth factor receptor, VEGFR, Mesoporous silica nanoparticles, MSNs, Angiogenesis

## Abstract

Anaplastic thyroid cancer (ATC) comprises approximately 2% of all thyroid cancers, and its median survival rate remains poor because of its resistance to conventional therapy. Vascular endothelial growth factor receptor (VEGFR)-targeted therapeutics-loaded mesoporous silica nanoparticles represent a major advance for angiogenesis imaging and inhibition in lethal cancers. In the present study, we aimed to assess whether ^131^I-labeled anti-VEGFR2 targeted mesoporous silica nanoparticles would have antitumor efficacy in an ATC tumor-bearing nude mouse model. Using in vitro and in vivo studies, we investigated the increased targeting ability and retention time in the anti-VEGFR2 targeted group using confocal microscopy and a γ counter. The tumor tissue radioactivity of the anti-VEGFR2 targeted group at 24 and 72 h after intratumoral injection was significantly higher than that of the non-targeted groups (all *P* < 0.05). Moreover, we found that radioactive accumulation was obvious even at 3 week post-injection in the anti-VEGFR2 targeted group via single-photon emission computed tomography/computed tomography, which was not seen at 3 day post-injection in the Na^131^I group. Meanwhile, compared with the non-targeted group, tumor growth in the targeted group was significantly inhibited, without causing apparent systemic toxic effects. Additionally, the median survival time in the targeted group (41 days) was significantly prolonged compared with that in the non-targeted (34 days) or Na^131^I (25 days) groups (both *P <* 0.01). Our data support the view that the as-developed ^131^I-labeled anti-VEGFR2 targeted mesoporous silica nanoparticles showed promising results in ATC tumor-bearing mouse model and such an approach might represent a novel therapeutic option for ATC.

## Background

Anaplastic thyroid cancer (ATC) only accounts for about 2% of all thyroid cancers; however, it is a locally aggressive type of tumor that has a high rate of distant metastases. Generally, the median survival of patients with ATC is about 6 months, with only 20% of patients surviving 1 year after diagnosis because of vascular catastrophe and airway collapse caused by aggressive regional disease invading neck tissues and lymph node and/or lung metastasis [1, 2]. Radioiodine-131 (^131^I) plays an important role in the diagnosis and treatment of differentiated thyroid cancer (DTC) metastases [3–5]. However, the conventional ^131^I treatment modality is not appropriate for ATC because of its non-iodine-concentrating characteristic [6].

Angiogenesis is a major factor contributing to the survival, growth, migration, and metastasis of cancer cells. Vascular endothelial growth factor (VEGF) is a crucial regulatory cytokine in angiogenesis, and plays an important role in the development of ATC. VEGF’s well-established role in angiogenesis means that radiolabeled ligands, such as bevacizumab, which targets the VEGF receptor (VEGFR), have been developed successfully for early and sensitive lesion detection using positron emission tomography (PET) and single-photon emission computed tomography (SPECT) imaging techniques. VEGFR-2 exerts most of the functions of VEGF in blood vessel endothelial cells, and is responsible for proliferation through the activation of the mitogen-activated protein kinase pathway, migration of endothelial vessels, and the promotion of angiogenesis and vascular growth [7, 8]. VEGF expression is upregulated in ATC cells from epithelial origin compared with that in normal thyroid tissue [9–11]. Anti-VEGF strategies have been developed to inhibit new blood vessel growth and starve tumors of necessary oxygen and nutrients. Several preclinical studies have developed drugs that target VEGFR2 in ATC with different degrees of success [12–15]. However, one of the major challenges related to these anticancer drugs is their low bioavailability and inefficient delivery to the target site. Many anticancer drugs are hydrophobic and need biocompatible drug delivery systems to improve their bioavailability and facilitate easier intravenous administration.

Nanoparticles, including metal-, polymer-, and lipid-based nanoparticles, can be used to combine a therapeutic agent with an imaging agent. Targeting ligands/moieties, such as peptides, antibodies, radionuclides, or antibody fragments, can be attached to nanoparticles to improve their therapeutic efficacy. For cancer treatment, nanoparticles could accumulate in the tumor area via an enhanced permeability and retention effect, with an ability to deliver therapeutic agents in a more localized fashion. For many years, in material sciences and engineering, silica has been considered as a versatile and relatively safe material because of the variety of available physical and chemical modifications that it offers, as well as its good biocompatibility [16]. The U.S. Food and Drug Administration (FDA) recognizes silica as “Generally Safe” [17, 18]. Among the various silica-based materials, mesoporous silica nanoparticles (MSNs) stand out as a class of nanomaterials with many distinctive advantages, such as their non-toxic nature, good surface permeability, high surface area, tunable pore structures, excellent physicochemical stability, and chemically modifiable surfaces, all of which make them potential hosts for various chemical agents and/or therapeutic drugs [19]. Similarly, mesoporous carbon nanomaterials have been demonstrated to be excellent nanocarriers in drug delivery [20–22]. In recent years, efforts in the field of MSNs have focused on combining therapeutic and imaging capabilities. Labeling nanoparticles with imaging probes is a valuable tool to enable their in vitro and in vivo tracking. Currently, targeted treatment modalities represent an encouraging strategy for ATC treatment [23, 24].

Inspired by the pivotal role of targeted therapy and molecular imaging against VEGFR in cancer research and the advantages offered by MSNs, in this study, we developed an anti-VEGFR2 targeting potentially theranostic nanoplatform based on surface engineering of MSNs for simultaneous noninvasive SPECT imaging and an in vivo enhanced ^131^I therapeutic effect. We aimed to determine whether ^131^I-labeled anti-VEGFR2 targeted MSNs would have antitumor efficacy in an ATC tumor-bearing nude mouse model, followed by extensive in vivo, in vitro, and ex vivo studies.

## Materials and Methods

### Materials

Dulbecco’s modified Eagle medium (DMEM), 0.25% Trypsin-EDTA, and fetal bovine serum (FBS) were purchased from Gibco (CA, USA). Antibiotics (penicillin G, streptomycin, and nystatin) were purchased from Dingguo Biotechnology Co. Ltd. (Beijing, China). Hexadecyltrimethylammonium bromide (CTAB), tetraethyl orthosilicate (TEOS), 3-aminopropyltriethoxysilane (APTES), dimethyl sulfoxide (DMSO), 1-(3-dimethylaminopropyl)-3-ethylcarbodiimide hydrochloride (EDC), *N*-hydroxysuccinimide (NHS), and fluorescein isothiocyanate (FITC) were purchased from Sigma-Aldrich (Germany). Na^131^I was purchased from Atomic Hitech (Beijing, China). Anti-VEGFR2 antibodies were purchased from Abcam Co. Ltd. (UK).

### Characterizations

The nanoparticles were dispersed in ethanol, forming a suspension, deposited on a carbon-coated copper grid, and dried for at least 24 h. The morphology of the nanoparticles was determined using transmission electron microscopy (TEM) (JEOL-100CXII, Japan) at an accelerating voltage of 200 kV. The zeta potentials and hydrodynamic diameters of the samples were measured using dynamic light scattering (DLS) using a Zetasizer (Nano ZS90, UK). The pore size distributions and surface areas of MSNs were characterized by Brunauer–Emmett–Teller (BET) and Barrett–Joyner–Halenda (BJH) analyses (ASAP2020M, USA).

### Cell Culture

The human ATC cell line FRO [25] (purchased from the Cell Resource Center, Institute of Basic Medical Sciences, Peking Union Beijing Medical College in Beijing, People’s Republic of China) was cultured at 37 °C in a humidified incubator with a mixture of 5% CO_2_ and 95% air and supplemented with 10% FBS and 1% penicillin–streptomycin. The medium was replaced every other day, and the cells were passaged by trypsin after confluence was reached. The cells were precultured until approximately 80% confluence was achieved before each experiment.

### Preparation and Surface Modification of Silica Nanoparticles

#### Synthesis of MSNs and Amination

The MSNs were synthesized as reported previously [26]. Briefly, 50 mg of CTAB was added to a mixture of 25 mL water, 5 mL ethanol, and 100 μL 2 M NaOH solution in a round-bottom flask under continuous stirring at 70 °C. Then, 200 μL of TEOS was added into the mixture and reacted for 1 h. The reaction solution was then centrifuged and washed with ethanol five times at 10,000 rpm. Next, the products were re-suspended in 10 mL of ethanol. After removal of the CTAB template, the product was then re-suspended in 5 mL of DMSO and 100 μL of APTES, which was added dropwise into the resultant mixture to modify the silica surface by amination. After overnight stirring, the precipitates were separated by centrifugation, washed with ethanol five times, and then the MSNs-NH_2_ were obtained.

#### Synthesis of BSA-MSNs-Anti-VEGFR2

Five milligrams of bovine serum albumin (BSA) and 50 μL of anti-VEGFR2 antibodies were added into the above product (MSNs-NH_2_, 50 mg) and reacted under stirring for 2 h. The mixture was washed with water five times, after which 1.1 mg of NHS and 1.6 mg EDC were added into the mixture and stirred overnight at room temperature in 5 mL of water. The precipitates were separated by centrifugation, washed with water five times, which successfully obtained the BSA-MSNs-anti-VEGFR2.

### ^131^I Radiolabeling of Nanoparticles

The produced nanoparticles were radiolabeled with ^131^I using the Chloramine-T method (denoted as ^131^I-BSA-MSNs and ^131^I-BSA-MSNs-anti-VEGFR2, respectively) [27]. Briefly, approximately 100 μg of BSA-MSNs or BSA-MSNs-anti-VEGFR2 were diluted with 100 μL phosphate buffer (PB), and 74 MBq ^131^I was added. Then Chloramine-T (100 μL; 5 mg/mL in PB) was added to the mixture. After 60 s of shaking and incubation, the reaction was stopped by adding 100 μL of sodium metabisulfite (5 mg/mL in PB). The centrifuge tube was used to separate labeled BSA-MSNs and BSA-MSNs-anti-VEGFR2 from low molecular weight compounds. The labeling rate and the radiochemical purity of ^131^I­labeled nanoparticles were determined using thin-layer chromatography.

### In Vitro Cellular Uptake: Confocal Microscopy Study

Firstly, the MSNs-anti-VEGFR2 and MSNs were labeled with FITC [28]. Briefly, MSNs-NH_2_ (100 mg) were added to 1 mL FITC alcohol solution (1 mg/mL), with standing for 4 h under stirring. FITC-labeled MSNs (FITC-MSNs) were obtained by centrifugation and dried in a vacuum. FITC-MSNs-anti-VEGFR2 could also be obtained using the same method.

FRO cells were seeded in 6-well plates overnight and then 5 × 10^5^ cells per well were incubated with FITC-MSNs and FITC-MSNs-anti-VEGFR2 for 1 and 6 h at 37 °C, respectively. Cells were washed three times with phosphate buffer saline (PBS) and then fixed with 70% ethanol for 20 min. Furthermore, the cells were washed three times with PBS and the nuclei were stained by 4,6-diamidino-2-phenylindole (DAPI) for 45 min and then fixed with paraformaldehyde (4% in PBS). Confocal microscopy images were acquired using a confocal laser scanning microscopy (CLSM) (Zeiss LSM 510, USA).

### Time-Dependent Cellular Uptake

To measure the time-dependent cellular iodine-131 uptake of the nanoparticles, 1 × 10^5^ cells per well were cultured with 3.7 MBq/mL free Na^131^I, ^131^I-BSA-MSNs, or ^131^I-BSA-MSNs-anti-VEGFR2, respectively. The cells were then washed as quickly as possible with 1 mL Hanks’ balanced salt solution (HBSS) buffer solution, detached with trypsin, and re-suspended in 1 mL HBSS for 1, 2, 3, 5, 7, and 9 h. Radioactivity was measured using a γ counter (LKB gamma 1261, Australia). All experiments were performed three times to obtain exact data .

### Animal Model

Balb/c nude mice (female, aged approximately 4 weeks, weighing 15–20 g) were purchased from the Beijing Experimental Animal Research Center at Peking Union Medical College and kept under specific pathogen-free conditions, with a relative humidity (30–70%) and temperature-controlled (20–24 °C) environment at the Laboratory Animal Center, Tianjin Medical University, China. All animal handling procedures were in accordance with a protocol approved by the Tianjin Medical University General Hospital Ethics Committee. FRO tumor xenografts were induced by subcutaneous injection of 5 × 10^6^ FRO cells in 50 μL of PBS into the right shoulder of the mice.

### In Vivo Imaging

When the tumor-bearing nude mice were fed for 1–2 weeks and the tumor volume had reached about 10 mm in diameter, the mice were randomly divided into three groups (Na^131^I, ^131^I-BSA-MSNs, and ^131^I-BSA-MSNs-anti-VEGFR2). Each group received 7.4 MBq ^131^I via intratumoral injection to assess the organ localization of ^131^I-labeled nanoparticles. Scintigraphic imaging was performed at 1, 2, 3, 7, 14, and 21 days after respective drug injection. Nude mice were anesthetized with 4% chloral hydrate (150 μL) before each scanning, placed in a prone position, and then imaged using a SPECT/CT scanner (Discovery NM/CT 670, USA). To avoid exposure of thyroid tissue to unwanted radiation and imaging, sodium perchlorate (0.05 mg/mL) was added to the drinking water for all mice 1 day before the experiment and maintained for 1 week.

### Tissue Distribution

At 24 and 72 h after intratumoral injection of 7.4 MBq ^131^I-labeled nanoparticles, the mice in each group (*n* = 3/group) were sacrificed by cervical dislocation, and the heart, spleen, kidney, liver, intestine, lung, and tumor tissues were removed and weighed. Radioactivity in various organs was measured using a γ counter (LKB gamma 1261, Australia), and the radioactivity was expressed as percentage of injected dose per gram of tissue (%ID/g).

### In Vivo ^131^I Therapy

Similar to in vivo imaging, the mice of the three groups were intratumorally injected with a dose of 74 MBq (50 μL) of ^131^I-BSA-MSNs-anti-VEGFR2, ^131^I-BSA-MSNs, and Na^131^I, respectively, when the tumor volume had reached about 10 mm in diameter. The same volume of normal saline was administrated as a control group. The tumor volume was estimated using the following formula: volume = 4π/3 (1/2 length × 1/2 width × 1/2 height) [15, 29]. The tumor volume and animal body weight were measured every 3 days. Mice were euthanized if they lost more than 20% of their body weight or became moribund.

### Histological Examination

When the experiment was over, the mice were sacrificed and the tumor and normal tissues of the four groups, including the heart, liver, spleen, kidney, and lung, were isolated to study their histopathology. Briefly, 5-mm-thick paraffin sections were dewaxed using two xylene incubations (for 30 min each at 56 °C) and then rehydrated in ethanol. The sections were then soaked overnight in 10% sucrose in distilled water. Thereafter, the sections were washed in 0.1 M PBS, pH 7.4, incubated in 1.2% hydrogen peroxide in methanol for 30 min, and rinsed in 0.1 M PBS, pH 7.4, for 15 min. The slides of the tumors were then incubated in a humid chamber overnight, at room temperature, with anti-VEGFR2 antibodies dilution 1:100. After washing three times with PBS, the slides were incubated with DAKO-REALTM En-Vision™ detection system for 60 min, then visualized using diaminobenzidine and counterstained with Mayer’s hematoxylin. All images were acquired using an Olympus microscope.

### Statistical Analysis

All data are presented as the mean ± standard deviation (SD), and statistical analysis was performed using SPSS (Statistical Package for Social Sciences) 12.0 for windows (SPSS, Chicago, IL, USA). Data analysis was performed using Kaplan–Meier curves and the log-rank test. All statistical tests were two-tailed, and a *P* value < 0.05 was considered statistically significant.

## Results

### The Characteristics of Nanoparticles

**Fig. 1:**
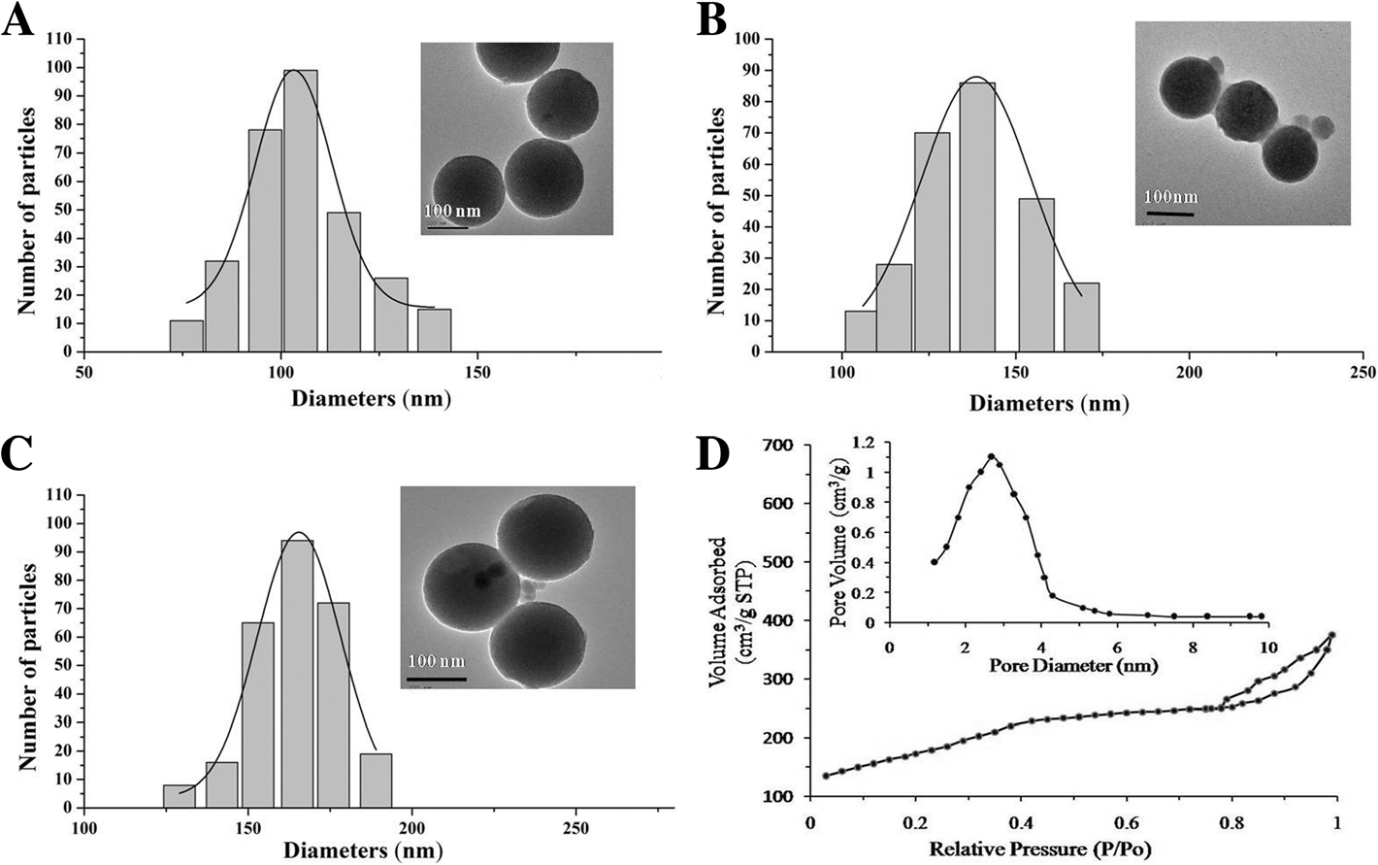
The characteristics of nanoparticles. Characterizations of MSNs (**a**), BSA-MSNs (**b**), BSA-MSNs-anti-VEGFR2 (**c**), and nitrogen adsorption-desorption isotherm and Barrett–Joyner–Halenda (BJH) pore size distribution curves of MSNs (**d**). The transmission electron microscopy images show that all had regular (uniform spherical) morphology. The dynamic light scattering images show that all were uniform, with average sizes of 108 nm, 139 nm, and 163 nm, respectively. BET and BJH analyses demonstrated typical type-IV isotherms, consistent with a mesoporous structure

The characteristics of nanoparticles were analyzed and determined by TEM and DLS (Fig. 1a–c). The TEM image demonstrated that the MSNs had uniform spherical morphology, and the DLS image showed that the MSNs were uniform with a size of 108 ± 5.9 nm. BSA modification and/or anti-VEGFR2 targeting did not change the morphology of the nanoparticles and only resulted in a slight tendency to aggregate in solution compared with unmodified MSNs, which may have been caused by the increased surface area of the nanocomplexes. The diameter of the BSA-MSNs-anti-VEGFR2 slightly increased to 163 ± 4.6 nm, and the mean zeta potential of MSNs was − 23.91 mV, which changed to 28.45 mV for BSA-MSNs-anti-VEGFR2. The change in zeta potential and the increase in size after surface modification suggested the successful addition of BSA and anti-VEGFR2 on the surface of the MSNs at each step (Table 1). The textural characterization of the MSNs was further confirmed by the nitrogen adsorption–desorption isotherm. The MSNs have well-defined mesoporous structure with a surface area of 630.2 m^2^/g, and an average pore diameter of 2.8 nm (Fig. 1d). The stability of BSA-MSNs-anti-VEGFR2 nanoparticles was monitored over several weeks, and no obvious aggregation observed. The proportion of ^131^I labeling was approximately 50–75%.

**Table 1:** Zeta potential and diameter of various particles

Sample	Zeta potential (mV)	Diameter (mm)
MSNs	− 23.91 ± 1.9	108 ± 5.9
BSA-MSNs	45.53 ± 1.6	139 ± 6.5
BSA-MSNs-anti-VEGFR2	28.45 ± 2.6	163 ± 4.6

### Uptake of Constructed Nanoparticles

**Fig. 2:**
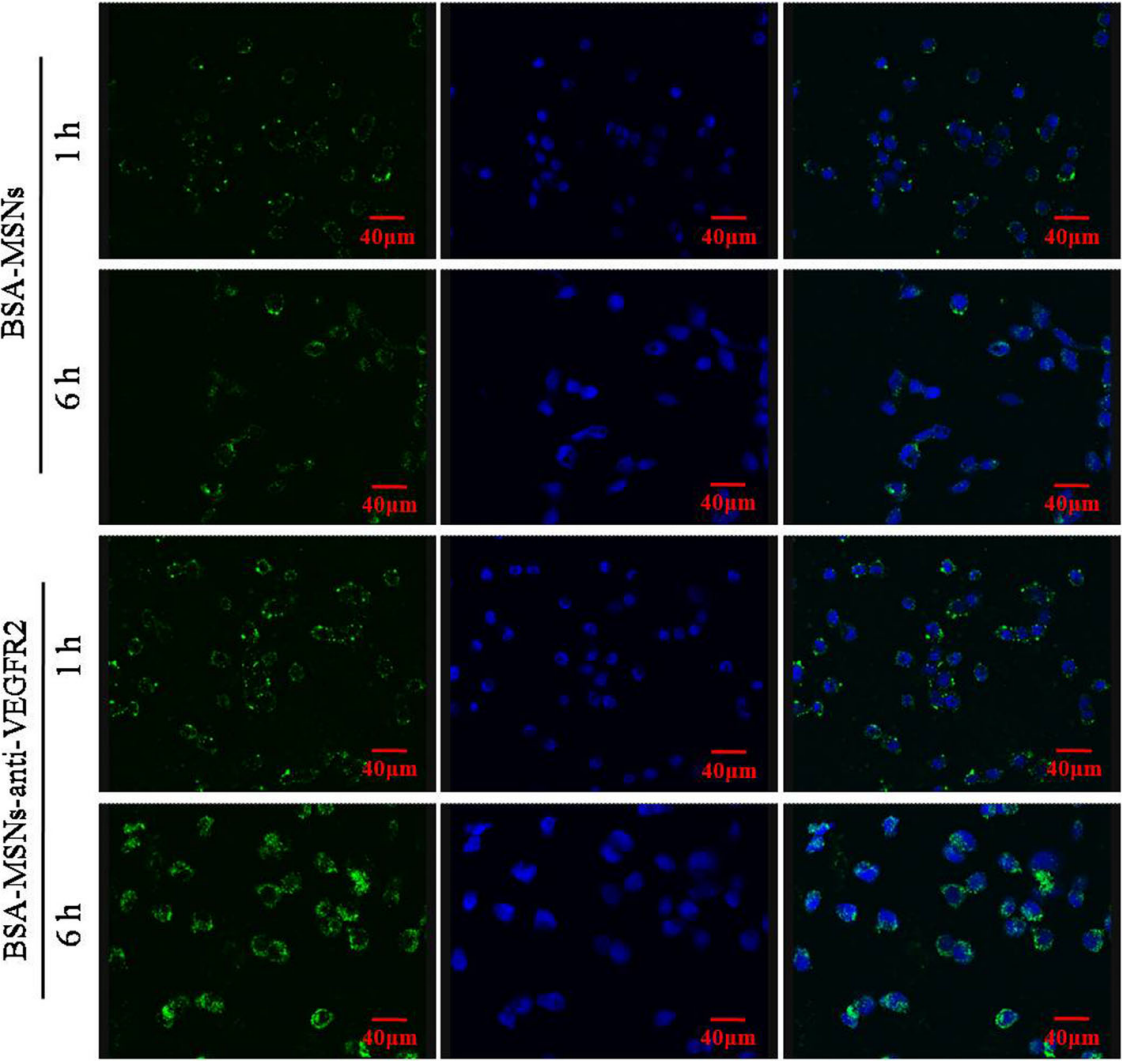
Uptake of constructed nanoparticles. Confocal laser scanning microscope images showed cellular internalization of different formulations at 1 and 6 h for BSA-MSNs and BSA-MSNs-anti-VEGFR2. The florescent signal at 1 h was increased at 6 h for both groups. Additionally, the binding green fluorescence in the anti-VEGFR2-targeted group was stronger than that in non-targeted group at both time points

The binding of BSA-MSNs and BSA-MSNs-anti-VEGFR2 to target human ATC cell line FRO cells was assayed using confocal microscopy (Fig. 2). Immunofluorescence showed that both targeted and non-targeted MSNs could efficiently bind to FRO cells. After 1 h of incubation with BSA-MSNs or BSA-MSNs-anti-VEGFR2, visible fluorescence was present in the cells, and which was maintained and strengthened after 6 h of incubation. Compared with BSA-MSNs-anti-VEGFR2, BSA–MSNs could also bind to cells, but we found that the tumor cell retention was minimal and the green fluorescence signal was weak. This result suggested that the targeting ability of the nanoparticles was enhanced via modification with the anti-VEGFR2 antibody.

### Time-Dependent Cellular Uptake

**Fig. 3:**
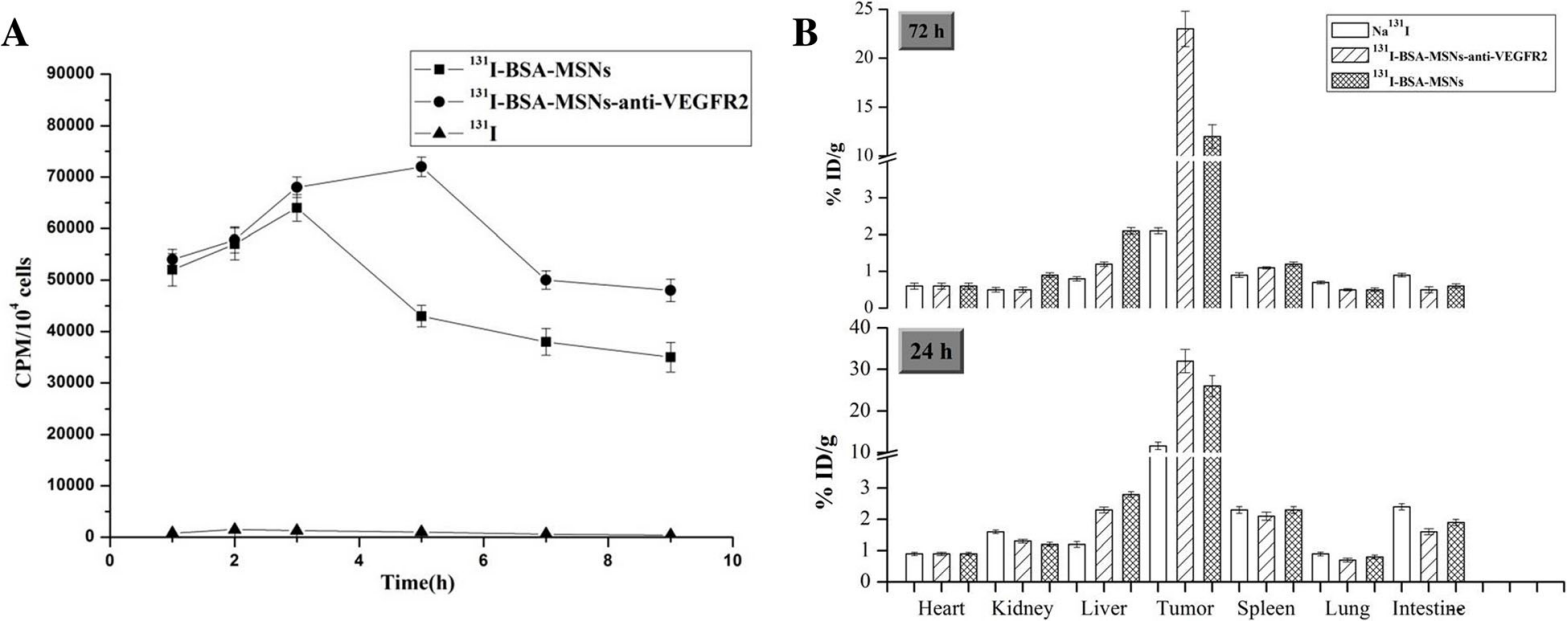
Time-dependent cellular uptake and tissue distribution. **a** The data on time-dependent cellular uptake are presented as the mean ± SD of three groups. **b** Comparison of the data on the biodistribution of ^131^I in the tumor and major organs of ATC tumor-bearing mice at 24 and 72 h after intratumoral injection of Na^131^I, ^131^I-BSA-MSNs, and ^131^I-BSA-MSNs-anti-VEGFR2. The tumor tissue radioactivity of anti-VEGFR2 targeted group at 24 and 72 h after intratumoral injection (32.2 ± 2.8% ID/g and 23.0 ± 1.8% ID/g, respectively) was significantly higher than that of non-targeted group (26.1 ± 2.5% ID/g and 12.3 ± 1.2% ID/g, respectively) (all *P* < 0.05). Data are also presented as the mean ± SD for the three groups

In order to determine the radioiodine uptake of Na^131^I, ^131^I-BSA-MSN, and ^131^I-BSA-MSNs-anti-VEGFR2 as time passed by, ^131^I-timed activity measurements were obtained in FRO cells. As shown in Fig. 3a, the ^131^I uptake in this cell line reached its maximal level after 3 h of incubation with ^131^I-BSA-MSNs and after 5 h with ^131^I-BSA-MSNs-anti-VEGFR2. Additionally, the radioiodine uptake of ^131^I-BSA-MSNs-anti-VEGFR2 was higher than that of ^131^I-BSA-MSNs.

### Tissue Distribution

The tissue distribution of ^131^I in nude mice was measured using a *γ* counter at 24 and 72 h after intratumoral injection with the respective drugs (Fig. 3b). The results revealed that the three groups had similar accumulation of radiation in the normal tissues at 24 and 72 h. The radioactivity for all groups gradually decreased with time and was mostly accumulated in the tumor. Additionally, the accumulation in the tumor tissue at 24 and 72 h post-injection in the two groups with ^131^I-labeled nanoparticles was much higher than that in the Na^131^I group, which was 11.6 ± 0.9% ID/g at 24 h and decreased dramatically to 2.1 ± 0.08% ID/g by 72 h. However, the concentration in the tumor of the anti-VEGFR2 targeted group at 24 and 72 h post-injection was 32.2 ± 2.8% ID/g and 23.0 ± 1.8% ID/g, respectively, which was significantly higher than that of non-targeted group (26.1 ± 2.5% ID/g and 12.3 ± 1.2% ID/g, respectively, all *P* < 0.05).

### In Vivo Imaging

**Fig. 4:**
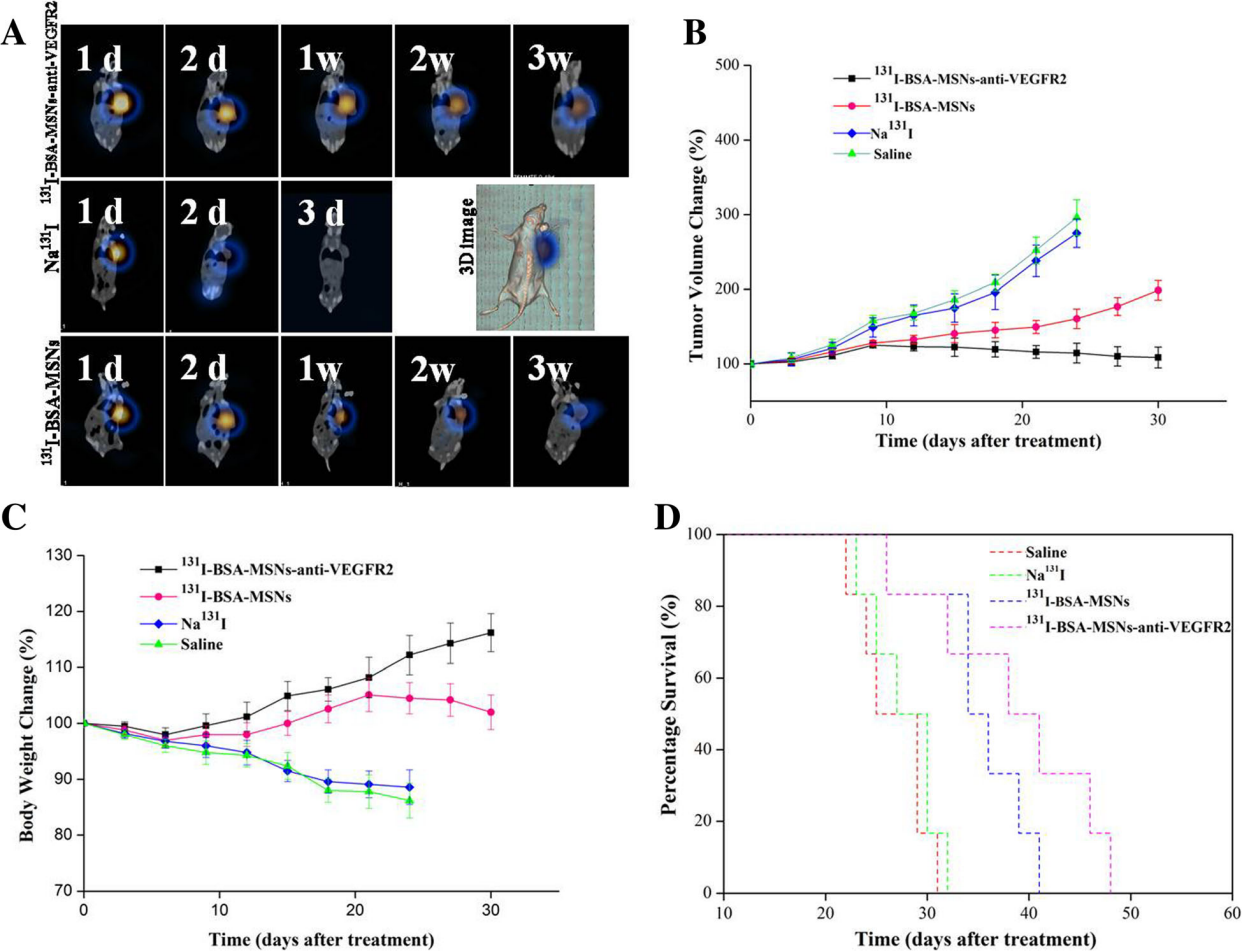
In vivo imaging, in vivo ^131^I therapy, and survival analysis. **a** SPECT/CT fused and three-dimension images of ATC tumor-bearing mice obtained at different time points after intratumoral injection with the respective drugs. The radioactive accumulation in the Na^131^I group was not seen at 3 days post-injection, but was obvious even at 3 weeks post-injection in the anti-VEGFR2 targeted group. Tumor volume (**b**), body weight (**c**) changes, and Kaplan–Meier survival curves (**d**) in the FRO ATC xenograft models (*n* = 6/group). Tumor growth and body weight loss in the targeted group were significantly inhibited compared with those in the non-targeted, Na^131^I, or saline group, respectively. Data are presented as the mean ± SD of three groups and all were *P <* 0.05. Additionally, the median survival time in the targeted group (41 days) was significantly prolonged compared with that in the non-targeted (34 days) or Na^131^I (25 days) group (all *P <* 0.01)

To determine the ^131^I uptake and distribution of nanoparticles and observe the residence time of ^131^I in the tumor tissue in vivo, representative SPECT/CT images of FRO tumor-bearing mice were acquired at different time points after intratumoral injection with the respective drugs (Fig. 4a). The results showed that the radioactive accumulation in the Na^131^I group was rapidly excreted from the tumor at 2 days post-injection, and was not seen at 3 days post-injection. By contrast, the two groups with ^131^I-labeled nanoparticles had slower blood clearance and higher accumulation in the tumor tissue even at 2 weeks post-injection, especially in the anti-VEGFR2 targeted group. Notably, at 3 weeks after injection, the radioactivity in the targeted group was obviously stronger than that in the non-targeted group.

### In Vivo ^131^I Therapy

Figure 4b shows the average tumor volume over time in the four groups. The tumor volume before injection was used as the initial reference. Except in the ^131^I-BSA-MSNs-anti-VEGFR2 group, the tumors gradually grew in all groups, especially in the Na^131^I and saline groups. On day 24, the mean tumor volume in the Na^131^I and saline groups was 296.6 ± 24.2% and 278.3 ± 19.3%, respectively, compared with 198.7 ± 13.2% in the ^131^I-BSA-MSNs group on day 30. Interestingly, we observed that the volume in the anti-VEGFR2 targeted group reduced slowly after day 9 post-injection and almost declined to the initial reference at the end of the observation.

Figure 4c shows the changes in the body weight of the four groups. The body weight gradually declined in the Na^131^I and saline groups during the observation period. However, the other two groups lost weight only in the first week, which was regained at later time points, especially for the anti-VEGFR2 targeted group.

### Survival Analysis

In this study, we used survival probability as another metric to evaluate the therapeutic effects of ^131^I-labeled nanoparticles. Figure 4d shows the Kaplan–Meier survival curves following treatments with saline, Na^131^I, ^131^I-BSA-MSNs, or ^131^I-BSA-MSNs-anti-VEGFR2 in the ATC tumor-bearing nude mouse model. Tumors progressed rapidly in the saline and Na^131^I groups, and the median survival time was 27 and 25 days, respectively. Analysis with a log-rank test revealed that the median survival time in the ^131^I-BSA-MSNs group (34 days) was significantly prolonged compared with that in the Na^131^I group (*P <* 0.001). Additionally, we found that the treatment in the anti-VEGFR2 targeted group (median survival time, 41 days) resulted in significantly better survival outcomes than those in the non-targeted group (*P* < 0.01).

### Histopathological Analysis

**Fig. 5:**
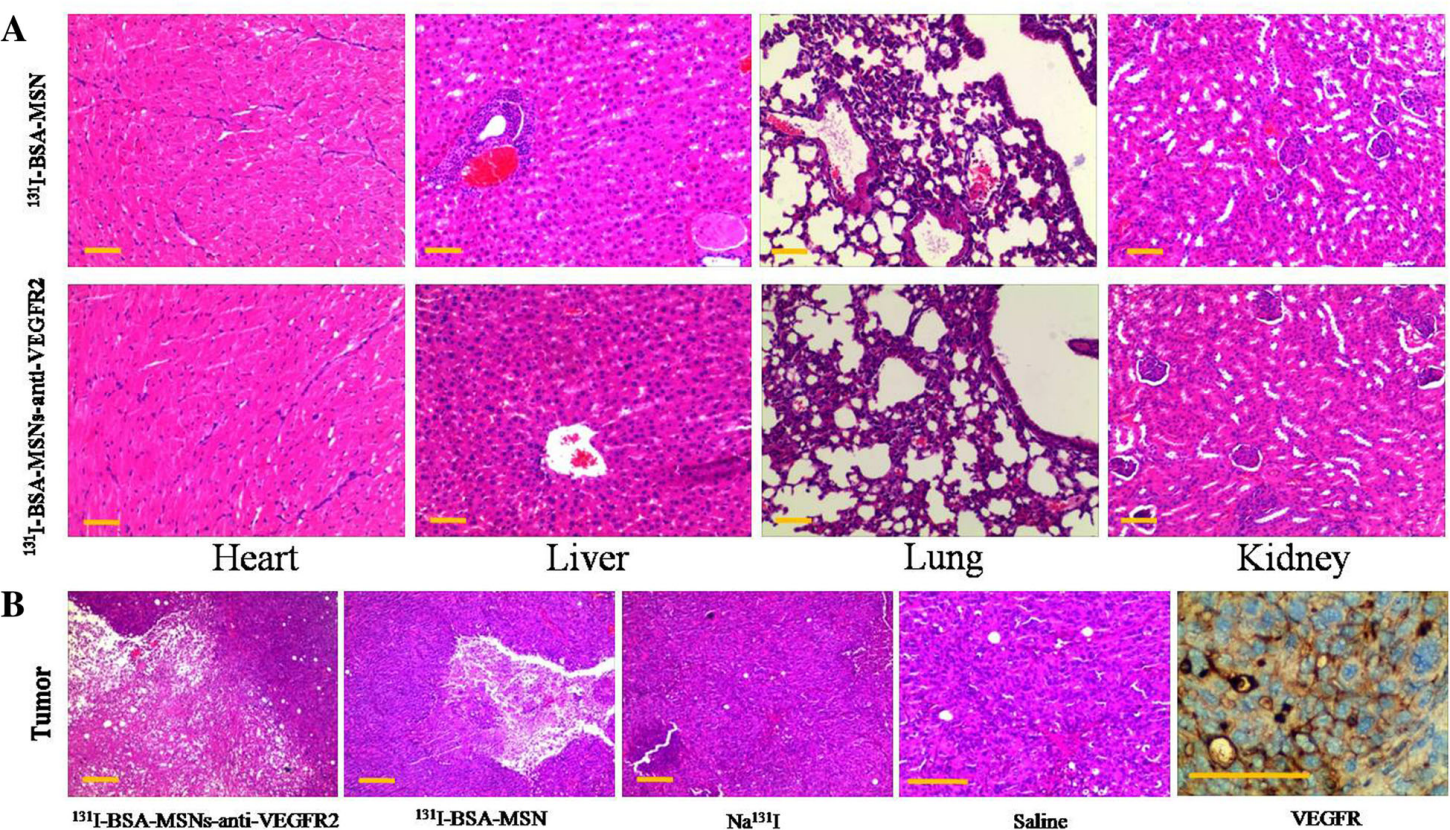
Histopathological analysis. **a** Histopathologic analysis of major organs of FRO ATC tumor-bearing mice after treatment with ^131^I-labeled nanoparticles. No significant pathological changes in the heart, liver, lung, and kidney were observed. **b** Pathologic examinations (H &E staining) and immunohistochemical analysis of tumors in mice. Large fragments from the degeneration and necrosis of tumor cells in the anti-VEGFR2-targeted group and small pieces of low density necrosis in the ^131^I-BSA-MSNs group are displayed. By contrast, only viable tumor cells were observed in the Na^131^I and saline groups. Photomicrograph from immunohistochemical analysis showed visible expression of VEGFR. Bar = 200 μm

To evaluate the antitumor effect of ^131^I-labeled nanoparticles and test the potential toxicity of MSNs in mice, the major organs and tumors were collected for hematoxylin and eosin staining after radiotherapy. No significant pathologic changes in the vital organs were observed in the tumor-bearing mice after treatment with ^131^I-labeled nanoparticles (Fig. 5a). This indicated that no apparent systemic toxicity had occurred within the period of observation. Additionally, the tumors treated with ^131^I-BSA-MSNs-anti-VEGFR2 showed degeneration and massive necrosis of tumor cells, which was more obvious than that in the ^131^I-BSA-MSNs group. However, the tumor treated with Na^131^I or saline group was packed with viable tumor cells. Immunohistochemical analysis of the tumor revealed visible expression of VEGFR (Fig. 5b).

## Discussion

The prognosis of ATC remains poor and there are no effective treatment options so far [1, 2, 6]. Targeted molecular therapy, as a novel therapy, has improved the morbidity and mortality of many cancers [23, 24]. VEGFR is crucial to microvascular formation, which facilitates the growth of most malignancies, and allows continued tumor expansion [9, 10]. In this study, we evaluated the efficacies of Na^131^I, ^131^I-BSA-MSNs, and ^131^I-BSA-MSNs-anti-VEGFR2 for the treatment of ATC in tumor-bearing nude mouse models. The results demonstrated that both anti-VEGFR2 targeted and non-targeted nanoparticles labeled with ^131^I were effective in delaying the tumor growth of ATC and that the ^131^I-labeling anti-VEGFR2 targeted MSNs was the most effective agent to inhibit the tumor growth in nude mice and prolonging median survival. This treatment modality might represent a novel therapeutic option for ATC.

VEGFR targeting with nanoparticles is rarely reported in the literature. Goel S et al. [19] confirmed that VEGFR targeting using VEGF_121_ conjugated, anti-VEGFR therapeutics-loaded MSNs represented a major advance for angiogenesis imaging and inhibition in human glioblastoma. A study performed by Gule indicated that inhibition of epidermal growth factor receptor (EGFR) and VEGFR2 in ATC using vandetanib causes significant tumor growth inhibition in vivo in an orthotopic xenograft model [15]. In the present study, using confocal microscopy, we found that both targeted and non-targeted nanoparticles could efficiently bind to the cytoplasm and cytoplast of FRO cells, and further confirmed that the targeting ability of the nanoparticles was enhanced via modification with the anti-VEGFR2 antibody, which result was consistent with that of the time-dependent cellular uptake experiment. Additionally, after intratumoral injection with the respective drugs, we compared data on the tissue distribution of ^131^I in the tumor-bearing nude mice at 24 and 72 h for the different groups. The radioactivity for all groups was mostly accumulated in the tumor and gradually decreased with time. Moreover, we found that the radioactivity in anti-VEGFR2 targeted group could be retained for longer time in the tumor in comparison with non-targeted group, which was also consistent with the results observed by confocal microscopy.

SPECT/CT is a powerful tool to provide both structural and functional imaging information for diseases, and can monitor the metabolism of radioactive drugs at different times post injection [30–32]. In the present study, we compared data on the tissue distribution of ^131^I using SPECT/CT. The results showed that radioactive accumulation in the Na^131^I group was not seen at 3 day post-injection. However, higher accumulation in the tumor tissue was observed at 2 weeks post-injection for the two groups with ^131^I-labeled nanoparticles, and at 3 weeks the radioactive signal in the anti-VEGFR2 targeted group was apparently stronger than that in the non-targeted group. We hypothesized that the passive tumor targeting of MSNs, which relies on unpredictable tumor extravasation and enhanced permeability retention effect, and positive targeting linked with anti-VEGFR-2, associated with VEGFR2 overexpression in ATC, played a key role in enhancing the retention of the nanoparticles in the tumors. This finding revealed that anti-VEGFR2 modification prolonged the retention time of ^131^I in the tumor tissue compared with that of free Na^131^I and ^131^I-BSA-MSN, which was also similar to the tissue biodistribution of ^131^I measured by *γ* counter.

In the present study, we monitored the body weight change of nude mice after intratumoral injection with a single dose of 2 mCi ^131^I, which showed that the body weight in the ^131^I-labeled nanoparticle groups gradually increased at 1 week post-injection, especially for the anti-VEGFR2 targeted group. Similarly, we also observed the changes in tumor volume and found that the tumors in the Na^131^I or saline group grew rapidly, while the volume in ^131^I-BSA-MSNs group increased slowly. Interestingly, the tumors in anti-VEGFR2 targeted group gradually decreased after 1 week post-injection, which was contrary to the body weight change. These results indicated that the anti-VEGFR2 modification could effectively inhibit the increase in tumor volume and thereby enhanced the efficiency of ^131^I therapy. We considered that the tumor necrosis was caused by the beta rays emitted from ^131^I, which resulted in tumor shrinkage and indirectly led to an increase in body weight. This effect, we speculated, began to appear mainly 1 week after injection of ^131^I.

Our findings indicated that the treatment mediated by intratumorally injected ^131^I-BSA-MSNs-anti-VEGFR2 resulted in significant tumor growth delay, which was confirmed increased structural damage and massive necrosis in tumor tissue compared with that in the ^131^I-BSA-MSNs group. Significantly, this higher antitumor activity was achieved without causing apparent systemic toxic effects, as indicated by the lack of significant pathological changes in the vital organs observed in tumor-bearing nude mice. Although further studies are needed to document both the acute and chronic toxicological effects, ^131^I-BSA-MSNs-anti-VEGFR2 exhibited several properties that made them a promising candidate for minimally invasive therapy for ATC.

In our pre-experiment, injection into the tail vein was performed and the results showed that the radioactivity was mainly distributed in the phagocytosis system. Intratumoral injection was used in some studies [29, 33, 34], and the operation is more convenient. Therefore, we used intratumoral injection as the injection method and also achieved better results. MSNs offer a promising approach to overcome the insolubility issue and deliver large payloads of hydrophobic small molecule drugs. Currently, we are investigating the potential for loading targeted anti-cancer drugs using MSNs. We will provide the results in the future publication.

## Conclusions

In the present study, we successfully synthesized BSA-MSNs-anti-VEGFR2, with uniform spherical morphology, and which were radiolabeled with ^131^I using the Chloramine-T method. The results showed that both targeted and non-targeted MSNs could efficiently bind to the cytoplasm and cytoplast of FRO cells. The radioactivity in ATC tumor-bearing nude mouse model was mostly accumulated in the tumor and could be retained a longer time in the ^131^I-BSA-MSNs-anti-VEGFR2 group. Additionally, the tissue distribution of ^131^I could be also imaged and validated using SPECT/CT. Moreover, tumor growth in the ATC tumor-bearing nude mouse model was significantly inhibited by the anti-VEGFR2 targeted MSNs compared with that achieved using non-targeted MSNs and the ^131^I treatment with anti-VEGFR2 targeting MSNs significantly prolonged the survival of ATC tumor-bearing mice. Our data supported the view that such an approach may represent a more effective means to treat ATC.

## Abbreviations

APTES: Aminopropyltriethoxysilane
ATC: anaplastic thyroid cancer
BSA: Bovine serum albumin
CLSM: Confocal laser scanning microscopy
CTAB: Hexadecyltrimethylammonium bromide
DAPI: 4,6-Diamidino-2-phenylindole
DLS: Dynamic light scattering
DMEM: Dulbecco’s modified Eagle medium
DMSO: Dimethyl sulfoxide
EDC: 1-(3-Dimethylaminopropyl)-3-ethylcarbodiimide hydrochloride
FBS: Fetal bovine serum
FITC: Fluorescein isothiocyanate
HBSS: Hanks’ balanced salt solution
MSNs: Mesoporous silica nanoparticles
NHS: *N*-hydroxysuccinimide
PBS: Phosphate buffer saline
TEM: Transmission electron microscope
TEOS: Tetraethyl orthosilicate
VEGFR: Vascular endothelial growth factor receptor
